# Sarcopenia diagnosis using skeleton-based gait sequence and foot-pressure image datasets

**DOI:** 10.3389/fpubh.2024.1443188

**Published:** 2024-11-27

**Authors:** Muhammad Tahir Naseem, Na-Hyun Kim, Haneol Seo, JaeMok Lee, Chul-Min Chung, Sunghoon Shin, Chan-Su Lee

**Affiliations:** ^1^Laboratory of Computer Vision and Human Visual Perception, Department of Electronic Engineering, Yeungnam University, Gyeongsan, Republic of Korea; ^2^Sport Science Major, School of Kinesiology, Yeungnam University, Gyeongsan, Republic of Korea

**Keywords:** sarcopenia, deep learning, convolutional neural network, spatio-temporal graph convolutional networks, foot pressure, skeleton

## Abstract

**Introduction:**

Sarcopenia is a common age-related disease, defined as a decrease in muscle strength and function owing to reduced skeletal muscle. One way to diagnose sarcopenia is through gait analysis and foot-pressure imaging.

**Motivation and research gap:**

We collected our own multimodal dataset from 100 subjects, consisting of both foot-pressure and skeleton data with real patients, which provides a unique resource for future studies aimed at more comprehensive analyses. While artificial intelligence has been employed for sarcopenia detection, previous studies have predominantly focused on skeleton-based datasets without exploring the combined potential of skeleton and foot pressure dataset. This study conducts separate experiments for foot-pressure and skeleton datasets, it demonstrates the potential of each data type in sarcopenia classification.

**Methods:**

This study had two components. First, we collected skeleton and foot-pressure datasets and classified them into sarcopenia and non-sarcopenia groups based on grip strength, gait performance, and appendicular skeletal muscle mass. Second, we performed experiments on the foot-pressure dataset using the ResNet-18 and spatiotemporal graph convolutional network (ST-GCN) models on the skeleton dataset to classify normal and abnormal gaits due to sarcopenia. For an accurate diagnosis, real-time walking of 100 participants was recorded at 30 fps as RGB + D images. The skeleton dataset was constructed by extracting 3D skeleton information comprising 25 feature points from the image, whereas the foot-pressure dataset was constructed by exerting pressure on the foot-pressure plates.

**Results:**

As a baseline evaluation, the accuracies of sarcopenia classification performance from foot-pressure image using Resnet-18 and skeleton sequences using ST-GCN were identified as 77.16 and 78.63%, respectively.

**Discussion:**

The experimental results demonstrated the potential applications of sarcopenia and non-sarcopenia classifications based on foot-pressure images and skeleton sequences.

## 1 Introduction

According to the 2021 population census data released by the National Statistical Office, people aged 65 years or older comprises 16.5% of Korean population (1). They are classified as an aging society according to United Nations standards. Thus, the average life expectancy is high, and according to the ‘OECD Health Statistics 2023’ published by the Ministry of Health and Welfare, the average life expectancy in Korea is 83.6 years, which is approximately 3 years greater than the OECD country average of 80.3 years. Therefore, health problems among the older adults have emerged as an important issue. In the 8th amendment to the Korean Standard Disease Sign Classification in 2021, sarcopenia was recognized as a disease, and the disease code (M62.5) was attributed to it.

Sarcopenia is a disorder in which skeletal mass, muscle mass, and function steadily decline owing to aging, inactivity, poor diet, and chronic illnesses (2, 3). The International Working Group on Sarcopenia (IWGS), the Asian Working Group for Sarcopenia (AWGS), and the European Working Group on Sarcopenia in Older People (EWGSOP) have varying definitions of sarcopenia. The official organization EWGSOP describes sarcopenia as a widespread skeletal muscle illness that progresses over time (3–5). Although the definition of sarcopenia varies slightly across institutions, a loss of muscle mass and physical performance is generally indicated along with the possibility for major health complications in the absence of proper treatment (6, 7). The combination of strength, endurance, power, and coordination determines physical function, which is crucial for maintaining musculoskeletal health. A wide range of musculoskeletal disorders (MSDs), ranging from acute injuries such as fractures and sprains to chronic illnesses such as rheumatoid arthritis and osteoarthritis, can result from aberrant muscle function in addition to sarcopenia (8).

Physical function, multiple sclerosis, and sarcopenia are intricately linked. Many tests are currently being conducted for physical function evaluation and early identification of possible problems with MSDs and sarcopenia. The chair stand exam, the timed up-and-go test, and the gait speed test are some of these assessments (9, 10). However, these techniques have some drawbacks as they can be affected by the subjectivity of the measurer and patient variability (11). Research to create smart gadgets that use pressure sensors, inertial measurement units (IMUs), and artificial intelligence (AI)-based measuring techniques are underway to extract pattern data from patients’ daily lives and overcome these restrictions (11–13). Smart insoles are commonly used to monitor patients with MSDs, including sarcopenia, by using pressure sensors and an IMU. They are particularly helpful for monitoring movement, detecting falls, and assessing balance. The pose estimation approach is also widely used in sports and gait analysis (14, 15).

Pose estimation is a cutting-edge computer vision technique that uses deep-learning models to estimate important human body parts instantly and reliably (16). Accurate 2D or 3D pose estimation enables the tracking and detection of body joints (17, 18). The efficacy and accuracy of pose estimation are being compared to those of the VICON motion system (Vicon Nexus; Vicon Motion Systems Ltd., Oxford, England), which uses numerous cameras to capture extremely accurate 3D motions (19, 20). Medical research aggressively investigates the assessment of physical functions, including the quest for precise body position tracking, while AI analytical tools continue to progress (11, 12). These developments have the potential to significantly improve both the diagnosis and treatment of a wide range of medical disorders, as well as our comprehension of human movement.

In this study, we first collected skeleton and foot-pressure datasets to effectively analyze gait for an accurate diagnosis of sarcopenia. Skeleton-based representation and foot-pressure exertion on the pressure plates is one of the methods that can effectively represent dynamic changes in human body movements and is widely used in human action recognition. Second, we performed rotation and flipping augmentations on the foot-pressure dataset for enhanced sarcopenia classification performance.

Our contributions can be summarized as follows:

- We collected foot-pressure and skeleton datasets from various populations and classified them into sarcopenia and non-sarcopenia groups based on grip strength, gait performance, and appendicular skeletal muscle mass (ASM) estimation, using the AWGS standard (Chen et al.).
- We performed flipping and rotation augmentations on the foot-pressure dataset using the ResNet-18 model to increase classification accuracy.
- We performed experiments on a skeleton dataset using the spatiotemporal graph convolutional network (ST-GCN) and demonstrated the potential applicability of sarcopenia classification from video sequences.

The remainder of this paper is organized as follows. Related work is summarized in Section 2. The datasets used in this study and the proposed models are described in Sections 3. Section 4 presents the experimental results and discussion, and Section 5 presents the conclusion of the study.

## 2 Related works

Owing to the recent advancements in machine and deep learning, these technologies are at the forefront of healthcare innovation, garnering considerable attention for their transformative impact on the diagnosis of conditions, such as gait disorders. By harnessing the power of intricate algorithms and neural networks, machine and deep learning not only enhance the accuracy and efficiency of diagnostic processes but also hold the promise of uncovering nuanced patterns and correlations within complex medical data. This section discusses various aspects of machine-learning and deep-learning methods to detect sarcopenia.

In Asian countries, the AWGS, an expert group, initiated a study on sarcopenia diagnosis in 2014. They identified three key diagnostic factors: muscle mass, muscle strength, and physical activity (gait). These diagnostic standards were revised in 2019 to address the limited accessibility of medical equipment such as MRI, CT, DXA, and muscle mass measurement tools. As an alternative, they proposed the use of bioelectrical impedance analysis and introduced a new category called the ‘possibility of sarcopenia.’ This category is used when muscle strength is low despite normal physical activity. The criteria for diagnosing the ‘possibility of sarcopenia’ or sarcopenia include handgrip strength less than 28 kg for men or less than 18 kg for women, or a 5-time chair stand test taking longer than 12 s. Sarcopenia is diagnosed when both ASM and muscle strength are low according to the aforementioned criteria. Severe cases where all three criteria were met were classified as severe sarcopenia (Chen et al.).

Logistic regression is a potent statistical method for predicting the risk and onset of sarcopenia in aging populations. Logistic models combine several factors, such as age, sex, physical activity, and body composition, to determine a person’s risk of developing sarcopenia (21–23). A noteworthy work by Kaur et al. (21) showed that logistic regression could be used to assess frailty and sarcopenia. Even with undefined variables, their model was remarkably accurate in predicting outcomes with a 97.69% prediction rate. To predict sarcopenia, Agnes et al. (22) created a multi-logistic model that included important factors such as BMI and calf circumference. With 80% sensitivity and 70% specificity, this model has the potential to be a useful screening tool. Yin et al. (23) combined logistic regression and nomogram visualization to accurately predict the risk of individual sarcopenia, highlighting its clinical utility.

Ko et al. (24) used the Support Vector Machine (SVM) analysis of data from inertial measurement devices during walking to achieve 95% prediction accuracy. Similarly, Kim created prediction models for sarcopenia by utilizing a variety of algorithms, including SVM, with public health data. The SVM model outperformed the LightGBM approach (25), yielding an accuracy of 85.2% in this investigation. Seok and Kim (26) highlighted data limitations as one of the main constraints. Sufficient datasets are a prerequisite for model training. Further details on the sensitivity of SVM to feature selection are provided by Kang et al. (27). Choosing the right features is essential to obtain the best possible prediction accuracy for sarcopenia. Castillo et al. (28) identified an important interpretability issue by drawing attention to the fact that SVM models are usually viewed as “black-box” models, which makes it difficult to understand how the model arrives at its predictions.

To facilitate generalization, random forest (RF) employs a large number of decision trees, each trained on bootstrap samples containing random elements (26). Sarcopenia modeling appears to benefit from the application of machine learning. Using 17 risk indicators, Kang et al. (27) compared the performance of RF with those of other classifiers in a specific application. Although logistic regression outperformed the other models, the 2000-tree RF model found significant predictors. Yoon et al. (29) used RF to predict the risk of sarcopenia in patients with cancer, whereas Seok and Kim (26) estimated the likelihood of sarcopenia in the older population using RF and physical activity data. RF is generally well suited for incorporating several clinical and anthropometric sarcopenia predictions because it is suitable for handling large datasets and nonlinear connections (26). Nevertheless, drawbacks, including subpar results for small datasets, challenges with missing data, and overfitting, exist.

To predict sarcopenia, gradient-boosting machines (GBMs) (26) have become versatile machine-learning tools. By combining multiple weak decision-tree models into one robust classifier, the GBM employs an ensemble technique. GBM introduces unpredictability while optimizing based on error metrics by repeatedly training models on a variety of activity data and other variables (26, 27, 30). With an AUC of 0.78–0.85, GBM yielded a prediction accuracy comparable to that of RF and logistic regression (26). Numerous useful elements were included, such as demographics (age and sex) (27), dietary characteristics (BMI and protein intake), and physical activity variables (gait speed, strength, and muscle mass) (26). Radiomics muscle characteristics acquired from CT scans also have potential (30). These diverse data facilitate the detection of the risk factors for sarcopenia. GBM models now incorporate activity-related characteristics such as BMI, walking speed, strength, muscle mass, and CT radiomics features (26, 30). These data have greatly aided the development of predictive sarcopenia frameworks to identify hazards in aging populations.

Gu et al. (31) presented an artificial intelligence body component measurement system (AIBMS) that uses deep learning to automatically divide body parts from abdominal CT scans and quantify the body component volumes and regions. Three network models, SEG-NET, U-NET, and Attention U-NET, were used in the development, and plain abdominal CT scan data were used for training. The segmentation model showed a high degree of accuracy, with a 0.9 DSC score for segmented body parts when tested using multi-device development and independent test datasets. Bae et al. (32) discussed a deep-learning model for forecasting a reduction in physical fitness caused by sarcopenia in individuals who may develop sarcopenia. Data from the Korean National Physical Fitness Award (2010–2023) were used in the study. The data comprised physical fitness and body composition indicators as well as exercise- and health-related assessments in Koreans aged >65 years. To characterize normal and potentially sarcopenic conditions, ASM was computed as ASM/height^2^ (33). The deep-learning model demonstrated 87.55% accuracy, 85.57% precision, 90.34% recall, and an F1 score of 87.89% to differentiate sarcopenia.

A long short-term memory (LSTM)-autoencoder-based anomaly detection system for orthopedic illnesses and its capacity to differentiate between normal and pathological gaits were presented in (34). To determine the gait characteristics of the human body, the sensitivity of the anomaly detection based on five human body points was analyzed. The diagnostic method identified 92% (*n* = 35) of the 38 individuals with sarcopenia. In addition to reduced physical functioning, a key factor in the diagnosis of sarcopenia is variations in gait disturbance performance among individuals who meet the criteria for sarcopenia (35). Physicians screen for sarcopenia by observing the patients’ habitual gait features without quantifying them. Such a subjective diagnosis has been considered problematic as different clinicians may arrive at different decisions because variables such as weariness may influence the diagnosis. A unique automated deep-learning model based on RF for real-time human body joint recognition was developed and paired with a modified LSTM to recognize gait parameters for additional clinical analysis and to enhance and facilitate the use of these data. The accuracy of the model was 90.9%. Wearable sensor data have also been used to predict various diseases. Chen et al. (36) presented a hardware- and software-based sarcopenia identification system, because alterations in human muscles mirror the symptoms of sarcopenia. The hardware consisted of multiple sensor modules (MSMs) and wearable devices used to gather electromyography (EMG) and gait signals. The software comprises a leg health classification net (LCNet) and a biomedical and inertial sensor algorithm.

A study that aimed to develop predictive and classification models for sarcopenia and discovered digital biomarkers is presented in (37). The method used plantar pressure data from 83 patients with smart insole equipment and a smartphone to collect video data for pose estimation. The Mann–Whitney U test was used to compare the data from 23 patients with sarcopenia and 60 patients in the control group. The physical capacities of patients with sarcopenia were compared with those of a control group by using a smart insole and pose estimation. Significant differences were found in 12 out of the 15 joint point variables analyzed; however, the knee mean, ankle range, and hip range did not indicate any differences. Another study presented a smart insole device and pose estimation based on AI, along with three classification models–RF, SVM, and artificial neural network—to classify control and sarcopenia groups (38). Approximately 67% of the patient data from 83 individuals were chosen for training, whereas the remaining data were split into test sets.

Medical image-based body part analysis can be used to detect sarcopenia. Thus, the development of an effective computational technique for disease prediction and autonomous body part segmentation is imperative. The work reported in (31) relates to the AIBMS for the diagnosis of the sarcopenia, which uses deep learning to automate the segmentation of body parts from abdominal CT scans and the quantification of body part areas and volumes. The work showed a high degree of accuracy with above 0.9 DSC score in segment body sections when tested using multi-device developing and independent test datasets.

We had previously proposed a single-model ST-GCN using an attention technique to classify pathological gaits from a skeleton dataset (39). We experimented with our model on the NTU RGB + D, GIST, and multimodal-gait symmetry (MMGS) datasets and obtained excellent performance. We enhanced our work by proposing single and multiple models to classify one normal and five pathological gaits (antalgic, lurch, steppage, stiff-legged, and Trendelenburg) using early and late fusion with publicly available foot-pressure and skeleton datasets (40). Foot-pressure data were fed into the transformer-based models, and skeleton data were fed into the ST-GCN. An evaluation was also conducted on single models and multi-model fusions, which involved applying early fusion to the feature vector and late fusion by combining the outputs from both modalities with and without different weights. The proposed single and multimodal models demonstrated good performance compared with those of other state-of-the-art methods.

Previous studies have focused on machine-and deep learning-based methods for the classification of sarcopenia. However, only a few studies have used deep learning-based methods. In addition, either skeleton or plantar foot-pressure datasets are focused on but not both. We have already proposed a single-as well as a multi-model-based system using the fusion of skeleton and foot-pressure datasets; however, it was for the classification of pathological gaits. Moreover, the size of the dataset was small, as deep-learning-based models require an adequate amount of data to achieve good performance. In addition, the data were not captured from real patients. Briefly, we need to capture a sufficient real-time dataset from patients using skeleton- and foot-pressure-based sensors. Furthermore, deep-learning-based models that can use skeleton-based and/or foot-pressure data are needed to classify sarcopenia. Table 1 contrasts various studies related to sarcopenia detection methods, highlighting the limitations of previous approaches and showcasing how our work, including the collection of a multimodal dataset (skeleton and foot-pressure), contributes to sarcopenia classification by experimenting on each modality separately.

**Table 1:** Comparison of related studies on sarcopenia prediction and classification with our contributions.

Reference	Description	Limitations	Our contributions
(21–23)	Studies on logistic regression models for predicting sarcopenia in aging adults.	Focus on traditional regression models and single predictive factors like muscle mass, lack of real-time data.	We collected a multimodal dataset (skeleton and foot-pressure), but experimented on each modality individually, applying deep learning models for sarcopenia detection.
(24, 25)	Machine learning classifiers to predict sarcopenia based on physical activity and physical factors.	Limited datasets and physical activity focus, lacks multimodal integration.	We experimented individually on skeleton and foot-pressure data from our collected dataset, offering higher accuracy through deep learning approaches.
(26, 27, 30)	Various machine learning approaches (SVM, RF, GBM) for sarcopenia prediction based on activity data.	Focus on specific datasets and machine learning techniques, lack of deep learning models or multimodal data.	We used deep learning (ResNet-18, ST-GCN) on skeleton and foot-pressure data individually, exploring their potential in sarcopenia classification.
(28, 29)	Automatic sarcopenia classification systems in hospitals and cancer patients.	Specific to hospital and cancer patients, limited generalizability.	We focused on a general older adult population using individual experiments on skeleton and foot-pressure data for broader applicability.
(31)	AI-based body part measurement system using CT scans for sarcopenia detection.	Relies on expensive CT imaging, which limits accessibility.	Our non-invasive approach analyzes skeleton and foot-pressure data independently, making it a more accessible option than CT-based methods.
(32, 33)	Deep learning models for predicting physical fitness and sarcopenia using health-related metrics.	Focus on physical fitness only and specific population (Korean), lacks multimodal approach.	We explored real-time gait and foot-pressure data individually, providing insights for sarcopenia detection without relying on fitness metrics.
(34, 35)	LSTM-Autoencoder anomaly detection for sarcopenia based on body joint composition.	Focus on anomaly detection and body joint analysis, no foot-pressure integration.	We separately experimented with foot-pressure and skeletal gait analysis to improve sarcopenia classification, without combining the data.
(36–38)	Use of wearable sensors and smart insoles for gait analysis to detect sarcopenia.	Focused on insole or sensor data alone, limited to a single modality.	We used our collected skeleton and foot-pressure data individually for sarcopenia classification, rather than relying on wearable sensors alone.
(31)	Deep learning system for body part segmentation using CT scans.	Focus solely on body part measures, lacking gait analysis.	Our study utilizes individual experiments on real-time gait and foot-pressure data for sarcopenia classification, offering a dynamic approach.
(39)	Early-Proposed single-model ST-GCN with attention techniques for pathological gait classification.	Focused on skeleton-based data, lacking integration with foot-pressure data.	By collecting our own dataset (skeleton and foot-pressure), we proposed a method for sarcopenia classification, using individual deep learning models for each data type.
(40)	Early-Proposed multimodal fusion models (early and late fusion) for gait classification.	Limited testing on publicly available datasets.	We collected our own dataset (skeleton and foot-pressure) and experimented on them separately for sarcopenia classification.

## 3 Materials and methods: skeleton-foot pressure-physical performance datasets for sarcopenia diagnosis by Yeungnam University (SF3PDB-YU) and proposed baseline models

This section provides a detailed overview of the materials and methods used in this study, which focuses on the dataset and the baseline models for sarcopenia diagnosis. Sequential skeleton data were collected using three Azure Kinect depth cameras (Microsoft, United States) to capture 3D skeletal information during gait sequences. Foot-pressure data were obtained using a pressure plate, allowing us to distinguish between normal and sarcopenic gaits. Additionally, physical performance metrics, such as grip strength and gait speed, were collected to assist in the diagnosis of sarcopenia. For the methods, we applied deep learning models, specifically ResNet-18 for foot-pressure data classification and Spatiotemporal Graph Convolutional Networks (ST-GCN) for skeletal sequence classification. The ResNet-18 model was fine-tuned on foot-pressure data to extract key features indicative of abnormal gaits, while the ST-GCN model was used to capture spatiotemporal relationships in skeletal sequences. Both models were evaluated for their ability to classify sarcopenia versus normal gait, using the collected datasets as inputs.

**Figure 1:**
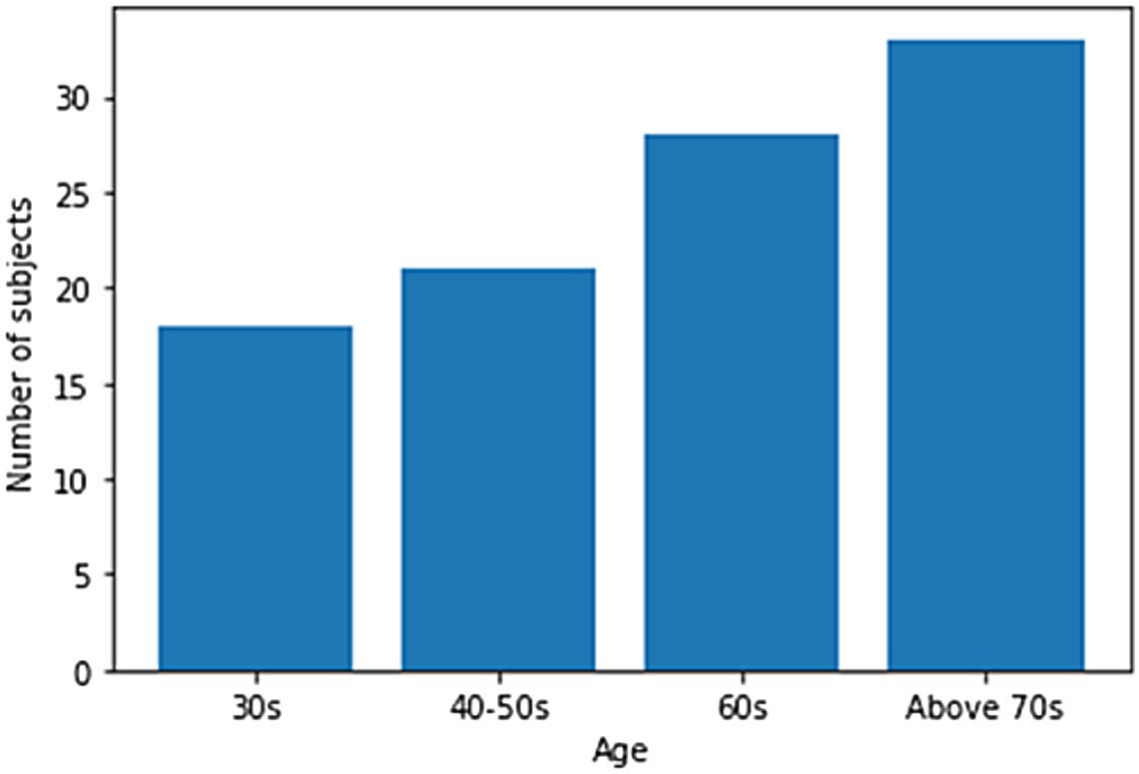
Age-wise distribution of participants for sarcopenia.

**Figure 2:**
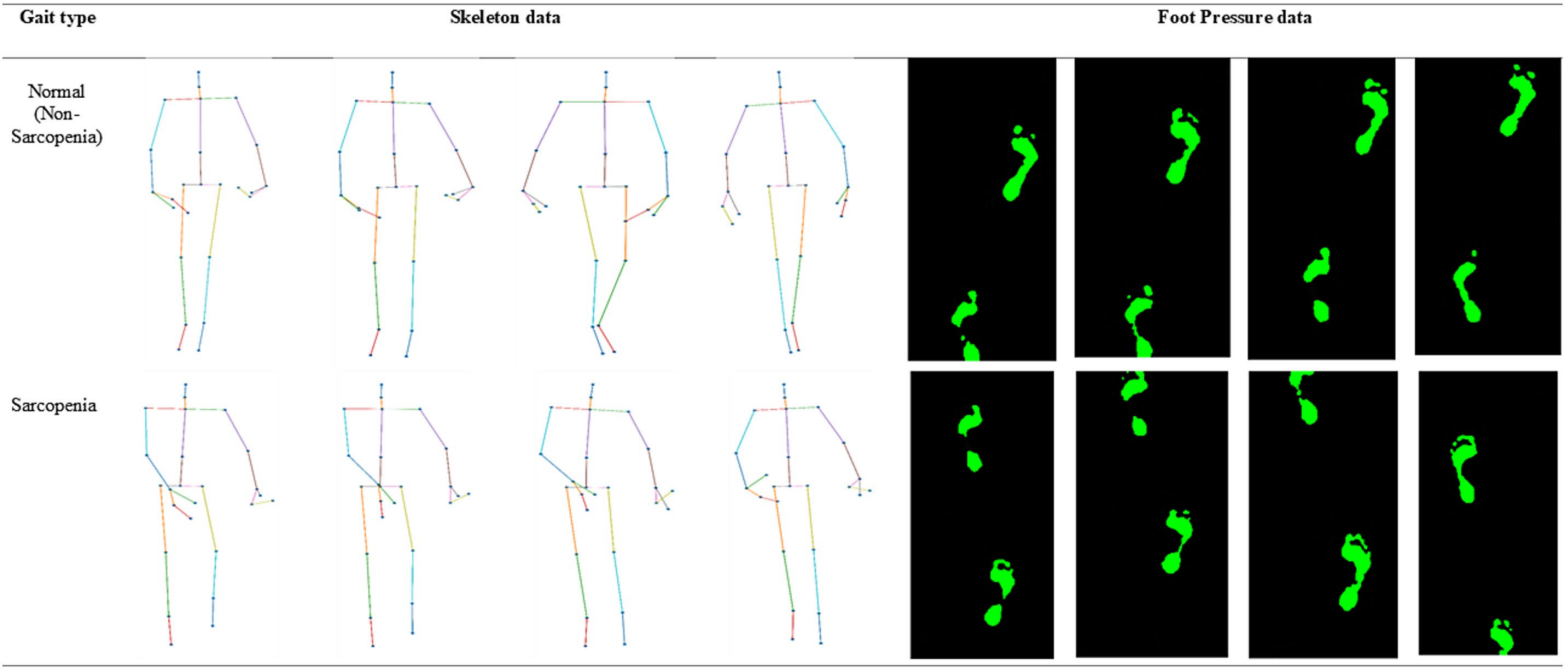
Representative samples of skeleton and foot-pressure data for normal and abnormal (sarcopenia) gait.

One hundred men and women with an average age of 55 years participated in the experiment. The number of men among the participants was 23, and the number of women was 77. All the participants agreed to use their data for research purposes. Figure 1 shows the age-wise distribution of participants. In the 30s age group, the number of patients was 18, whereas in the 40–50s, the number of patients was 21. Similarly, in the 60s age group, the number of patients was 28, while in the age group of 70 years and above, the number of patients was 33. The number of participants above 70 years of age was higher than that in other age groups. Data collection was conducted under strict supervision, and when participants walked through a 6-meter walkway, sequential skeleton and foot-pressure data were collected. A few samples of normal and abnormal (sarcopenia) gaits from the skeleton and foot pressure data are shown in Figure 2. The skeletal data and foot-pressure data in Figure 2 illustrate clear distinctions between normal (non-sarcopenia) and sarcopenia gaits. In the skeleton data, the sarcopenia group demonstrates more restricted joint movement, particularly in the hips and knees, leading to shorter and uneven strides. The joint angles in sarcopenia subjects indicate a reduced range of motion, which affects their overall gait efficiency. In contrast, the non-sarcopenia group shows more extended joint movements, with fluid and coordinated strides, allowing for a more efficient walking pattern. In the foot-pressure data, the pressure distribution differs significantly between the two groups. Non-sarcopenia subjects exhibit a more even pressure distribution across the entire foot, especially in the heel and forefoot regions, which are critical for balanced walking and effective propulsion. On the other hand, sarcopenia subjects show reduced pressure on these key areas, particularly in the forefoot, indicating weaker muscle strength and a diminished ability to push off effectively during walking. This imbalance in pressure contributes to an unstable gait pattern and increases the risk of falls in sarcopenia subjects. Additionally, the foot-pressure data for sarcopenia subjects reveals a more staggered and irregular footprint pattern, further demonstrating instability and compensatory gait behaviors, such as reduced stride length and slower gait speed. These combined observations in both skeletal and foot-pressure data highlight the biomechanical differences between normal and sarcopenic gaits, underscoring the impact of muscle weakness and joint stiffness in sarcopenia.

### 3.1 Skeleton data collection

**Figure 3:**
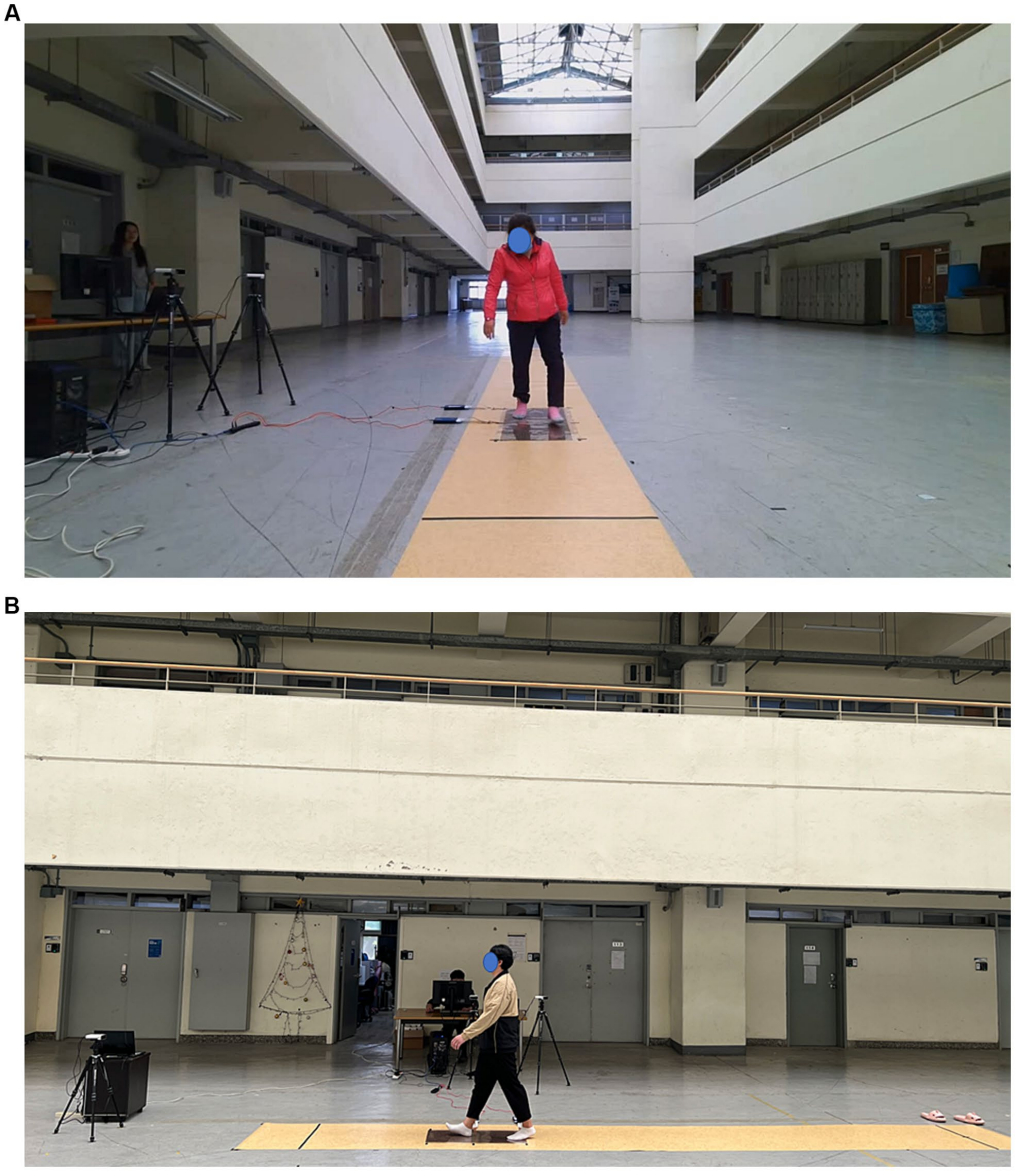
System setup for multimodal data collection system: (A) front view and (B) side view.

The Azure Kinect is the latest released Kinect sensor. We obtained the skeleton data using the Azure Kinect sensor and the corresponding Microsoft software development kit (SDK). Figures 3A,B show our multimodal setup views for data collection from the front and side, respectively, where we collected the skeletal gait data of the walkers on a 6-meter walkway. We obtained the 3D xyz coordinates of 33 joints, i.e., pelvis, spine_naval, spine_chest, neck, clavicles, shoulders, elbows, wrists, hands, hand tips, thumbs, hips, knees, ankles, feet, head, nose, eyes, and ears. The x- and y-axes represent the width and height of the participant, respectively, and the z-axis represents the distance between the camera and participant. The participant walked 6 m back and forth 10 times using the gait performed in daily life, and the frontal view of the participant walking was recorded in an RGB + D video using an Azure Kinect camera.

#### 3.1.1 Selection of 25 joints from 32 joints

**Figure 4:**
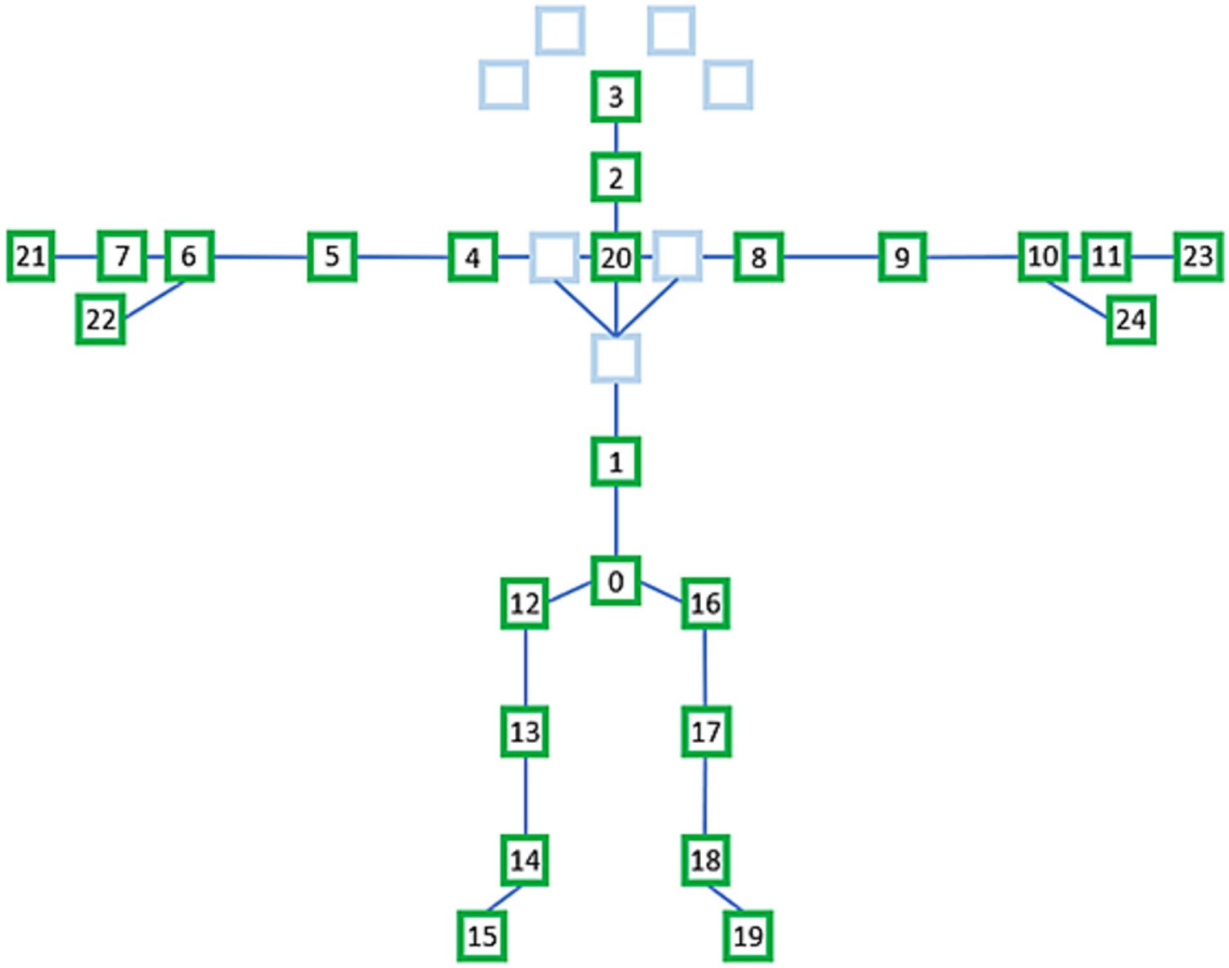
Selected 25 joints from 32 joints.

We extracted the data for 25 joints from that of the 32 joints for each frame, as shown in Figure 4. The names of the selected 25 joints shown in Figure 4 are as follows: 0—sacrum, 1—center of the spine, 2—neck, 3—head, 4—left shoulder, 5—left elbow, 6—left wrist, 7—back of left hand, 8—right shoulder, 9—right elbow, 10—right wrist, 11—back of right hand, 12—left hip, 13—left knee, 14—left ankle, 15—left foot, 16—right hip, 17—right knee, 18—right ankle, 19—right foot, 20—center of the shoulder, 21—left middle finger, 22—left thumb, 23— right middle finger, and 24—right thumb.

### 3.2 Foot-pressure data collection

A 6-meter walkway was set up, and a force plate was installed in the middle. Here, we used the average foot pressure, which was calculated by averaging the planar foot pressures for all time sequences. As shown in Figure 2, the average foot pressure can be expressed as a 1-channel image. We collected gait datasets for 100 people who moved back and forth 10 times; therefore, the expected total number of sequences was 100 × 10 × 2 = 2,000. However, some of the collected sequences contained missing data and were excluded from the evaluation. Consequently, 1986 skeleton and foot-pressure instances were obtained.

To ensure the accuracy of measurements for both the left and right feet, as well as to accommodate different step lengths among participants, the foot-pressure data was processed in the following way:

**Data Filtering and Cleaning**: Foot-pressure sequences with significant measurement errors or missing data were excluded to maintain data quality. Only sequences with reliable foot-pressure data for both the left and right feet were included in the final dataset.

### 3.3 Physical performance tests for sarcopenia

Several performance tests for sarcopenia are used in the literature like hand-grip strength (HGS), short physical performance battery (SPPB), gait speed, timed up and go (TUG), and five times sit-to-stand tests (41). In this study, the HGS and gait speed were measured according to the AWGS criteria for sarcopenia. To assess the gait speed of individuals, researchers have utilized a 6-min walking test. This test is highly correlated with the functional performance of older adults and has been established as a valid and reliable measurement method. During the test, the participants engaged in self-paced walking within an enclosed gymnasium. To track gait speed, the participants wore two inertial measurement unit sensors specifically attached to the insteps of their shoes. In this study, only data from the right foot were analyzed to measure the gait speed. To demarcate the turning point, cones were placed at both the starting point and the 30-meter mark. Throughout the test, the participants continuously walked back and forth within a 30-meter distance for a total of 6 min. In addition, the power-grip strength of the participants was measured three times using a hand dynamometer (hydraulic pinch gauge, Jamar, United States). The average of the measured values was used as the final value. The power grip was measured in a sitting position, and the width was adjusted such that the second joints of the four fingers, excluding the thumb, were at right angles when the grip strength measurement sensor was held with the elbow joint bent at 90°.

#### 3.3.1 Hand-grip strength

Traditionally, HGS has been quantified using measurement techniques and dynamometers. Recently, novel techniques for measuring grip strength have been proposed. To evaluate hand-grip strength, Jeong et al. (42) used the joint angles of the fingers from finger tracking. This method has an error rate of less than 15% and has the potential to be converted into a mobile application. Additionally, Barrios et al. (43) presented a straightforward mobile application that measures the speed at which fingers tap, which is a measure of grip strength.

However, grip strength determined by these techniques varies, leading to a broad variety of cutoff positions for sarcopenia screening (44, 45). Moreover, grip strength varies across nations and is predicted to affect the prevalence of sarcopenia. The frequency of low HGS and sarcopenia, as well as their prognostic usefulness for physical performance, were thus strongly influenced by the choice of the HGS criterion (average vs. maximum) (46). Similarly, De et al. (47) contended that HGS, an independent predictor of sarcopenia, should be used as a screening instrument to divide the population for whom confirmatory CT-based evaluation of sarcopenia is necessary.

Sarcopenia diagnosis requires standardization of HGS measures and criteria to comprehend variations in grip strength by nation, age, and sex. As a result, numerous studies have attempted to establish cutoff points and standardize the measurement of grip strength. To standardize the measurement of grip strength (48), Roberts et al. (49) proposed the “Southampton protocol,” and Schaap et al. (50) performed a systematic review. Notwithstanding these efforts, several variables can still affect grip strength and, thus, the cutoff points for diagnosing sarcopenia, including race, body size, lifestyle variations, and socioeconomic status (51).

**Figure 5:**
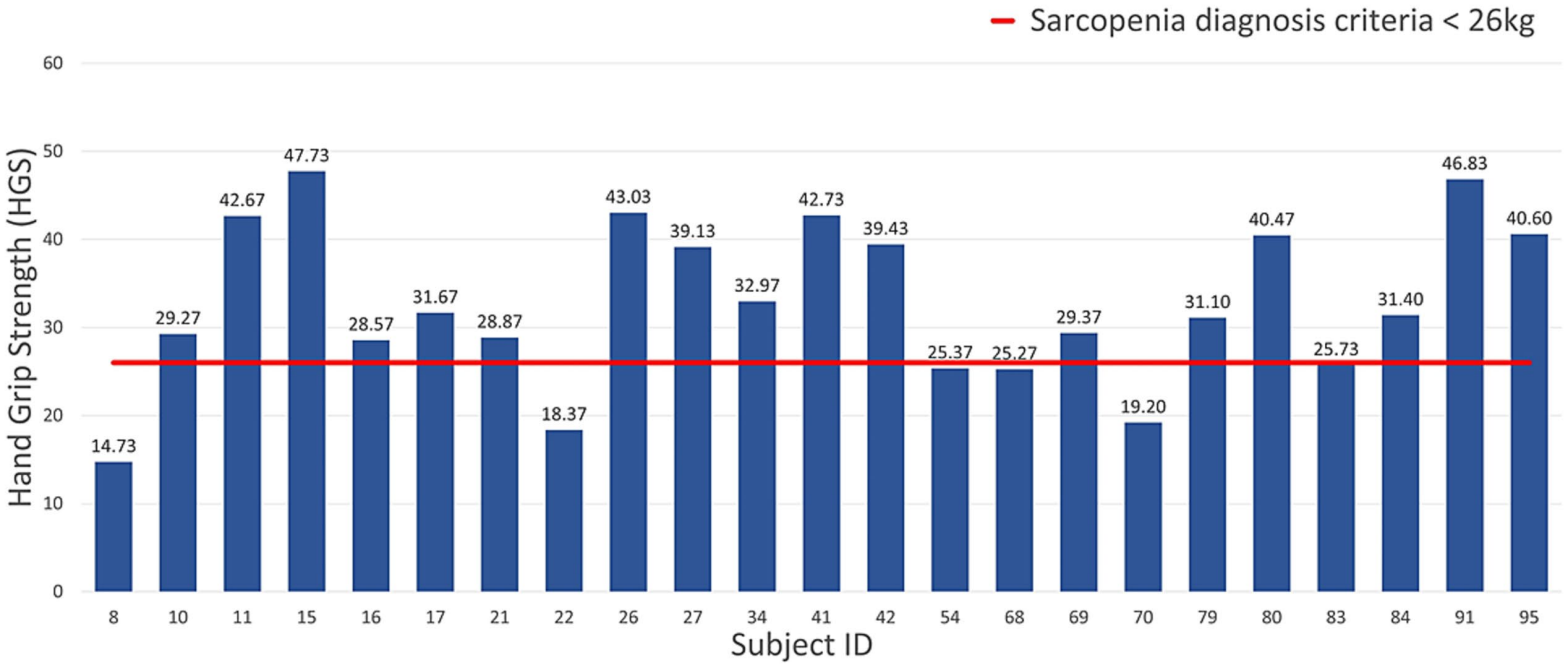
HGS of female participants using the criteria for sarcopenia diagnosis.

**Figure 6:**
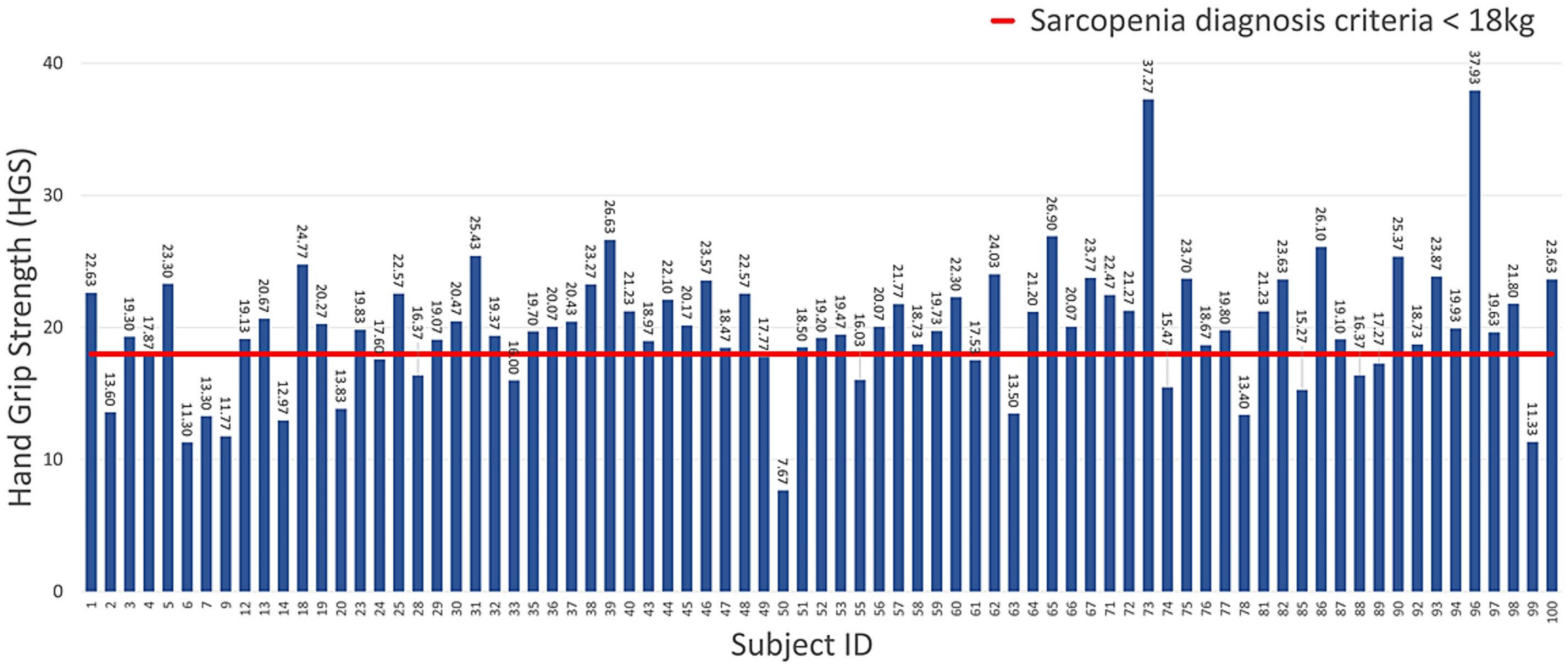
HGS of male participants using the criteria for sarcopenia diagnosis.

The gaits of men and women were labeled as normal and abnormal, according to the AWGS 2019 sarcopenia diagnostic criteria for HGS (sarcopenia for men weighing less than 26 kg, sarcopenia for women weighing less than 18 kg) and others. Figures 5, 6 show the measured HGS values of female and male participants, respectively, with sarcopenia according to the AWGS 2019 diagnostic criteria.

### 3.4 Methods: proposed baseline models

In this section, we first present the single model ResNet-18 for our foot-pressure dataset, then present the ST-GCN for our skeleton dataset, and finally, discuss the normalization of the skeleton dataset. Both models were used to classify sarcopenia.

#### 3.4.1 ResNet-18 for foot-pressure dataset

**Figure 7:**
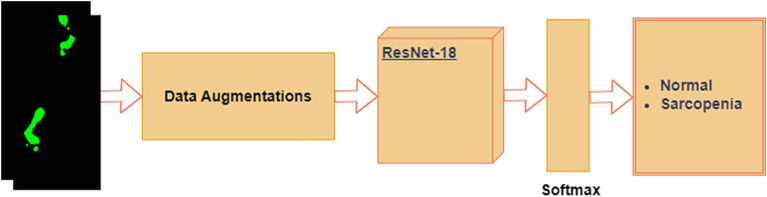
Proposed single modal ResNet-18 for the foot-pressure dataset.

It is possible to train deep neural networks (NNs) with more than 150 layers using residual neural networks (ResNet). By introducing residual blocks (RB) (52), ResNet can address the issues of vanishing gradients and deterioration (53) caused by the constant increase in CNN depth. Residual blocks create a “skip connection” that fast-forwards to a deeper layer by adding the output from the previous layer to the layer above. In this study, we fine-tuned the pre-trained ResNet-18 model for the foot-pressure dataset, leveraging the benefits of residual connections to prevent vanishing gradients. A softmax layer was placed at the end of the ResNet-18 architecture to classify images into either “normal” or “sarcopenia,” as shown in Figure 7.

The steps in the process are outlined below:

1. **Data Augmentation**: Given the limited training data, we applied data augmentation to increase the dataset size and prevent overfitting. For each foot-pressure image, the following transformations were applied: flip/mirror the image with a probability of 0.5 and rotate the image at an angle of 45°.
2. **ResNet-18**: After data augmentation, the images were passed through the pre-trained ResNet-18 model, which was fine-tuned for our foot-pressure dataset. ResNet-18, with its residual blocks, helps manage the vanishing gradient problem and allows for efficient training, even with a deep network.
3. **Softmax Layer**: At the end of the ResNet-18 architecture, the softmax layer provides a probability distribution across the two classes, “normal” and “sarcopenia.” The final classification is based on the highest probability.

In this study, we chose ResNet-18 as the CNN model for foot-pressure data classification for several reasons. First, ResNet-18 employs a residual learning framework, which addresses the vanishing gradient problem commonly encountered in deeper networks. This is particularly beneficial for capturing the complex spatial patterns in foot-pressure data over time. Furthermore, ResNet-based models have shown strong generalization abilities in gait and action recognition tasks, closely related to our focus on sarcopenia classification. ResNet-18’s lightweight architecture, compared to deeper variants like ResNet-50, also ensures efficient training and inference, making it ideal for real-time classification. Additionally, its compatibility with transfer learning allows us to fine-tune the model on our relatively smaller dataset, leveraging pre-trained features to enhance performance while reducing the risk of overfitting.

#### 3.4.2 ST-GCN for skeleton dataset

Data from successive time series comprise skeleton sequence data. The use of graph convolutional networks (GCNs) in skeleton-based action detection has significantly increased (54, 55). The ST-GCN-based model displayed strong generalization abilities and increased expressive power. Pathological gait categorization has recently used the ST-GCN, combining 3D skeletal data with attention technique (56). This novel method enables focus on important joints in the current gait by introducing an attention mechanism for spatiotemporal GCNs. There are two types of edges: temporal edges, which link the same joints over successive time steps, and spatial edges, which follow the inherent connections of the joints. In addition, several ST-GCN layers were built, enabling information integration along the temporal and spatial dimensions. This is accomplished by first using joint angles to extract spatiotemporal information from 3D skeletal data and then applying these features to GCNs.

##### 3.4.2.1 Normalization of skeleton dataset

The x-axis of the 3D skeleton data represents the left side direction of the participant, the y-axis represents the participant’s up side direction, and the z-axis represents the distance between the participant and camera. The range of the x-, y-, and z-axis coordinate values of the 3D skeletal data varies depending on the participant’s height, which may affect learning. To conduct efficient and accurate learning, it is necessary to reduce the height differences between participants. The 3D skeletal data coordinate values were normalized based on the distance from the 0-sacral vertebrae to the 20-shoulder center on the x-, y-, and z-axes of each frame. We used Equation 1 to determine the distance (d) from 0-sacral vertebrae to the 20-shoulder center in three-dimensional space. For each of the 25 joints, the x, y, and z-axis coordinate values were normalized by dividing the distance (d) using Equations 2–4, respectively.

(1) $$d=\sqrt{{\left(x_{0}-x_{20}\right)}^{2}+{\left(y_{0}-y_{20}\right)}^{2}+{\left(z-z_{20}\right)}^{2}}$$

(2) $$x_{\mathrm{scaled}\_i}=\frac{x_{i}}{d}$$

(3) $$y_{\mathrm{scaled}\_i}=\frac{y_{i}}{d}$$

(4) $$z_{\mathrm{scaled}\_i}=\frac{z_{i}}{d}$$

##### 3.4.2.2 Sarcopenia classification by the normalized skeleton sequences

**Figure 8:**
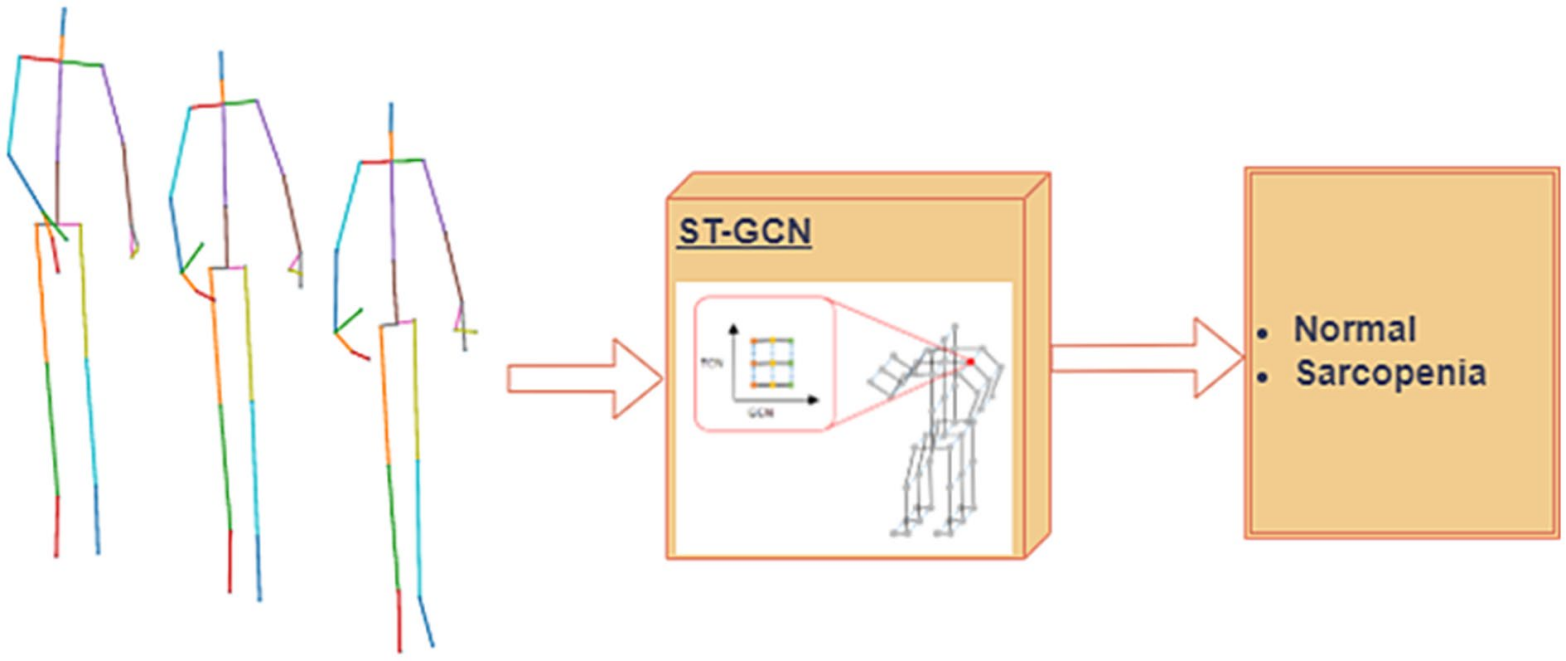
Proposed single modal ST-GCN (39) for the skeleton dataset.

When classifying pathological gaits using skeletal data, the ST-GCN attention mechanism makes it easy to focus on important joints. A block diagram of the classification of the normal and sarcopenia classes is shown in Figure 8. Here, we applied our previously suggested ST-GCN model (40), which uses an attention technique applied to pathological gait classification based on skeleton information. When implementing this model, we had two main goals. The process is outlines as,

1. **Spatiotemporal Feature Extraction**: The first goal was to extract spatiotemporal features from the skeletal sequence data, which are represented as graphs. The nodes of the graph correspond to body joints, while the edges represent the joint connections (spatial edges) and the temporal relationships between joints across time steps (temporal edges). These features are crucial for capturing the dynamics of gait.
2. **Attention Mechanism**: The second goal was to introduce an attention mechanism into the ST-GCN model, which allows the model to focus on the most important joints during the classification process. This mechanism helps the model learn which joints are most relevant to identifying sarcopenia-related gait abnormalities.
3. **Multi-Input Feature Integration**: In addition to joint positions, we integrated multi-input features such as joint velocity and bone orientation to improve classification accuracy. This multi-branch approach has been validated through ablation studies, which demonstrated the contribution of each component to the overall performance of the model.

## 4 Results: experimental results of the baseline models

To assess the performances of the proposed single- and multimodal classification models, a leave-one-subject-out cross-validation approach was employed. We used a four-fold cross-validation to assess the performance and generalization ability of the model. The number of participants with sarcopenia was 20; therefore, we divided them into four folds (five participants in each fold), while the number of participants in the normal (without sarcopenia) class was 80; therefore, we divided them into four folds (20 participants in each fold). Thus, we created four folds, with 25 participants in each fold [5 participants in the sarcopenia class and 20 participants in the non-sarcopenia (normal) class]. During the training, each fold was used separately for each test. Consequently, the model was trained four times, and the accuracies were averaged for all folds. During training, we separated 20% of the training dataset (three participants in the sarcopenia class and 12 participants in the non-sarcopenia class) for validation.

For the foot-pressure dataset, a learning rate of 0.001 and the Adam optimizer were used. We used cross-entropy as the loss function, and the entire experiment was conducted over 100 epochs. For the skeleton dataset, we used a learning rate of 0.1 and an SGD optimizer with a momentum of 0.9. We also used the learning rate of each parameter group using a cosine annealing schedule (57) with a weight decay of 0.001 and cross-entropy as a loss function. The experiment was conducted over 200 epochs. We used an Intel (Santa Clara, CA, United States) CPU with 32 GB of RAM and an NVIDIA (Santa Clara, CA, United States) GeForce RTX 3060 GPU for the evaluation. The models used in this study were implemented using the PyTorch software.

The selection of hyperparameters, such as the number of epochs, learning rate, and optimizers, was based on a combination of best practices from related studies and empirical tuning for our specific datasets. For the foot-pressure dataset, we found that 100 epochs provided sufficient time for the model to converge, while for the skeleton dataset, 200 epochs were necessary due to the complexity of spatiotemporal data. The learning rates (0.001 for ResNet-18 and 0.1 for ST-GCN) were chosen to ensure stable and efficient convergence. Additionally, we used the Adam optimizer for ResNet-18 due to its adaptive learning capabilities, and the SGD optimizer with momentum for ST-GCN, as it is known to perform well with spatiotemporal models. These settings, combined with weight decay and cross-entropy loss, helped optimize the performance of both models.

Table 2 lists the accuracies of the foot-pressure dataset using the ResNet-18 model and the skeleton dataset using the ST-GCN model. For fold 1, the accuracies of ResNet-18 and ST-GCN were 77.22 and 80.12%, respectively. For fold two, the accuracies of ResNet-18 and ST-GCN were 76.68 and 79.95%, respectively. For fold three, the accuracies of ResNet-18 and ST-GCN were 86.89 and 80.08%, respectively, whereas for fold four, the accuracies of ResNet-18 and ST-GCN were 67.87 and 74.39%, respectively. The average accuracies of the ResNet-18 and ST-GCN models were 77.16 and 78.63%, respectively.

**Table 2:** Accuracies of ResNet-18 model using the foot-pressure dataset and ST-GCN model using the skeleton dataset.

Four fold cross-validation	ResNet-18	ST-GCN
Fold 1	77.22%	80.12%
Fold 2	76.68%	79.95%
Fold 3	86.89%	80.08%
Fold 4	67.87%	74.39%
Average	77.16%	78.63%

## 5 Conclusion

In this study, we present our collected foot-pressure and skeleton datasets. Although the original skeleton dataset consisted of 32 joints, only 25 joints were used in each frame. To reduce the height differences between participants, the 3D skeletal data of each frame were normalized based on the distance from the sacral vertebrae to the shoulder center. The normalized 3D skeletal time-series data were labeled as sarcopenia and non-sarcopenia gait based on the participant’s physical performance, according to the AWGS 2019 guidelines. Second, we experimented with the foot-pressure dataset for the ResNet-18 model and the skeleton dataset for the ST-GCN model using 4-fold cross validation; the presence or absence of sarcopenia was predicted with an average accuracy of 77.16% for the foot-pressure dataset and 78.63% for the skeleton dataset.

This study is highly relevant to public health and aging populations, as sarcopenia is a prevalent condition among older adults that leads to decreased muscle mass, strength, and physical performance. Early diagnosis and intervention can significantly improve quality of life and reduce healthcare burdens associated with falls, frailty, and disability in aging individuals. By developing a system that can classify sarcopenia based on foot-pressure and skeletal data, this study provides a foundation for accessible and effective diagnostic tools that can be used in clinical and home settings, potentially contributing to better management of age-related diseases.

In future, we plan to extend this research by increasing the number of participants and improving the accuracy of sarcopenia classification. Moreover, in the future, we can develop a real-time system for the quick and efficient diagnosis of sarcopenia using a camera. We will also attempt to improve the overall performance by improving the spatiotemporal attention mechanism. Because the model uses multiple inputs, lightening of the model is also an important concern; we plan to apply an appropriate lightweight deep learning techniques for practical application in an edge device, while maintaining its performance.

## Data Availability

The datasets presented in this study can be found in online repositories. The names of the repository/repositories and accession number(s) can be found at: https://github.com/Hasaren/Sarcopenia-Gait-Dataset.
